# Salutogenic nursing home care: Antonovsky’s salutogenic health theory as a guide to wellbeing

**DOI:** 10.1093/heapro/daad017

**Published:** 2023-04-14

**Authors:** Sigrunn Drageset, Sidsel Ellingsen, Gørill Haugan

**Affiliations:** Institute for Health and Caring Sciences, Faculty of Health Social Sciences, Western Norway University of Applied Sciences, Bergen, Norway; Faculty of Health Sciences, Department of Nursing, VID Specialized University, Bergen, Norway; Department of Global Public Health and Primary Care, Faculty of Medicine, University of Bergen, Bergen, Norway; Department of Public Health and Nursing, Norwegian University of Science and Technology, Trondheim, Norway; Faculty of Nursing and Health Science, Nord University, Levanger, Norway

**Keywords:** sense of coherence, wellbeing, nursing home residents, palliative care

## Abstract

The nursing home (NH) population is characterized by a high symptom burden resulting from chronic illnesses and functional impairments that cannot be cured. Most long-term NH residents are in the last phase of life and in need of palliative care. Hence, health and wellbeing are important aims of salutogenic NH care, which includes more than the treatment of residents’ diseases and symptoms. Research shows that cognitively intact long-term NH residents with a high score on sense of coherence (SOC) experience better wellbeing. Therefore, NH care should be developed in a salutogenic direction, promoting residents’ health and wellbeing by identifying general and specific resistant resources and facilitating residents’ perceived SOC. Based on Antonovsky’s salutogenic health theory and focusing on SOC comprising comprehensibility, manageability and meaningfulness along with resistance resources, this article discusses how nurses can apply salutogenic knowledge as a guide to promote wellbeing among long-term NH residents.

## INTRODUCTION

Age is not an illness; however, illness and diseases increase with age. Therefore, most chronically ill people are older. A high incidence of chronic illness and functional impairments (Hoben *et al.*, 2016) results from increased longevity; thus, several older adults are in need of nursing care at home or in a nursing home (NH). In Norway, the policy is to allow older adults to stay at home as long as possible (Meld. St. 26, 2014–2015). Consequently, a move to an NH results from numerous losses, illnesses, disabilities, loss of functions and social relations and facing the end of life, all of which increase an individual’s vulnerability and distress. The scope of Norwegian NH care includes long-term care, palliative care (PC), care for those with dementia or physical disabilities along with relief and care for family members caring for older people with dementia or physical disabilities at home. This means that NH care includes both terminal (dying well) and long-term residential care as well as short-term care for rehabilitation and family alleviation. Hence, the main goals of NH care are quality of life (QoL), wellbeing and a good death, which include more than treating residents’ diseases and symptoms (Haugan, 2014c; Haugan et al., 2016, 2020b). Currently, about 40 000 people are living in Norwegian NHs (Statistics Norway, 2020); the mean age is about 86 years, and the mean residential time is 2 years. However, residential time varies; in several municipalities, 35% of NH residents die within 6 months (Norwegian Directorate of Health, 2020). In Norway, about 40% of all deaths annually occur in NHs (Statistics Norway, 2020). Consequently, many NH residents are in their last phase of life and should be acknowledged as palliative patients requiring different types of medical and nursing care (Hoben *et al.*, 2016). The long-term NH population is characterized by a high incidence of chronic illness and functional impairments (Hoben *et al.*, 2016), multiple simultaneous and complex diagnoses with a severe symptom burden, impaired functioning, various losses and fewer social relationships (Haugan, 2014b; Söderbacka *et al.*, 2017; Rinnan *et al.*, 2022). Typically, NH residents are characterized by frailty and vulnerability; pain, fatigue, dyspnoea, constipation and insomnia are common symptoms (Haugan, 2014c; Rinnan *et al.*, 2022), and depression, anxiety and loneliness are common psychological ailments (Brownie and Horstmanshof, 2011; Beerens *et al.*, 2013; Drageset *et al.*, 2013; Hoben *et al.*, 2016; Erdal *et al.*, 2017; Drageset and Haugan, 2021). Moreover, many NH residents struggle with existential issues, such as ‘what makes my life worth living’ and ‘how do I cope with the finality of my life’. Struggling with such issues can result in existential suffering and distress (Grech and Marks, 2017). The few studies available show the need for NH residents to talk about existential issues (Haugan, 2013, 2014b,c,d; Smedbäck *et al.*, 2017; Sjöberg *et al.*, 2018). In contrast, although perception of existential suffering is emphasized in research on cancer care and palliative medicine (Kissane, 2012; Gautam *et al.*, 2019), this has rarely been studied in the NH context. Consequently, this population is at high risk of emotional and physical distress along with declining purpose and meaning in life (Sanderson and Scherbov, 2010; Steptoe *et al.*, 2015). Alternative approaches to increase wellbeing and perceived meaning in life among older adults in NHs are needed. Spirituality, including perceived purpose and meaning in life, hope, self-transcendence and existential wellbeing, has been seen as a key resource for wellbeing in later life (Kane, 2003; Daaleman *et al.*, 2008; Fegg et al., 2010, 2014; Hedberg *et al.*, 2011; Steptoe *et al.*, 2015; Martela and Steger, 2016; Czekierda *et al.*, 2017; Duppen Rn *et al.*, 2019; Man-Ging *et al.*, 2019). Research implies that perceived meaning in life is important for maintaining not only mental/emotional wellbeing but also physical and functional wellbeing (Haugan, 2014b,c). According to Norwegian national guidelines (Helsedirektoratet, 2019), all NH wards must provide a service that meets the residents’ needs for basic alleviating treatment and palliative care (PC) (Svendsen *et al.*, 2017). The vision of NH care is that palliative care is woven into the fabric of NH practices, reflecting our perspective that PC is an inseparable component of high-quality NH care (Froggatt, 2001; Ersek *et al.*, 2022).

The main goal of the modern hospice philosophy is to facilitate wellbeing and meaningfulness until death (Saunders, 2006). The World Health Organization defines the concept of healthy ageing as ‘the process of developing and maintaining the functional ability that enables wellbeing in older age’ (World Health Organization, 2021). This also applies to older individuals staying in NHs, despite their disabilities and symptom severity. World Health Organization defines PC as an approach improving QoL and wellbeing among individuals (World Health Organization, 2019). This means that the goals for NH care and PC coincide. PC prevents and relieves suffering by means of early identification of ailments, thorough assessment and treatment of pain and other problems. A current trend is to view PC as an approach that should be integrated in a standard of nursing care in general (Coyle, 2015; World Health Organization, 2019). This trend challenges what is seen as specific to PC as opposed to basic nursing care. In an NH context, PC includes a holistic approach to relieve ailments and enhance residents’ wellbeing (Haugan, 2014b,c, 2021a; Sekse *et al.*, 2018; Rinnan *et al.*, 2022). Saunders introduced the concept of ‘total pain’ including physical, mental, social and spiritual/existential aspects, which are interwoven and cannot be viewed separately (Saunders, 2006). She therefore emphasized an interdisciplinary approach and awareness that also involve concern for the family. Hence, integrating the palliative approach as an essential aspect of NH care requires a ‘total pain’ perspective that also includes the family. Long-term NH residents are in the last phase of life and in need of PC (Emilsdóttir and Gústafsdóttir, 2011).

Wellbeing is seen as a resource for health and illness; wellbeing is more than health and can be found within illness, disabilities and the finality of life. Regardless of disease, infirmities and facing death, focusing on an individual’s wellbeing directs attention towards the person, his/her experiences here and now, and what is on the person’s mind (Snowden *et al.*, 2018). Accordingly, focusing on wellbeing provides opportunities for alleviation of suffering, thus promoting wellbeing and health when living with chronic illness and functional impairments.

The main goal of PC is that the individual should be able to live as well as possible despite health challenges. In a palliative approach, a core question is: ‘What is important to you?’, or ‘What is on your mind?’ The answers to these questions provide direction for the care, which should be adapted to what makes sense in the specific life situation as well as to what the individual masters, understands and handles. The salutogenic health theory may serve as a guide to promote wellbeing in NHs.

### Salutogenesis: the salutogenic health theory

Beyond a more traditional, pathogenic focus on risks, diseases and problems, the salutogenic approach focusing on aspects that support health and wellbeing. The World Health Organization Ottawa Charter clearly defines health as a ‘… a resource for everyday life … A positive concept emphasizing social and personal resources, as well as physical capacities ….’ Salutogenesis represents a resource-oriented theoretical approach; the focus is on the origin of health along with people’s abilities and capacity to function well and wellbeing. In general, the salutogenic approach involves an area of knowledge and learning, a way of relating to others, and a way of working in a health-promoting manner (Haugan and Eriksson, 2021b). The salutogenic viewpoint embraces health as a movement on a continuum between ease and disease. Consequently, no one is categorized as solely healthy or diseased; we are all somewhere between the imaginary poles of total wellness and total illness, including NH residents. Hence, directing wellbeing is essential independently of where within this continuum the individual is moving. The salutogenic health theory developed by Antonovsky (Antonovsky, 1987) involves three central concepts: sense of coherence (SOC), general resistance resources (GRRs) and specific resistance resources (SRRs). These concepts influence each other.

Antonovsky defined SOC as:

A global attitude that expresses the extent to which one has a pervasive, lasting but also dynamic sense of confidence that (1) the stimuli that come from one’s inner and outer environment are structured, predictable and understandable; (2) sufficient resources are available for one to cope with the demands of these stimuli; and (3) these requirements are challenges worth engaging. [(Antonovsky, 1987), p. 37]

The SOC construct comprises three components: (i) comprehensibility, (ii) manageability and (iii) meaningfulness. *Comprehensibility* expresses the degree to which the individual experiences and understands the impressions he/she receives from the environment. Individuals with a high degree of understanding are more likely to expect that what happens in the future is understandable and predictable, instead of chaotic and random. If events happen surprisingly, these will still be comprehensible, explained and put into context (Antonovsky, 1987).

*Manageability* describes the degree to which the individual experiences having resources available to meet challenges and difficulties; that is, all resources that the individual has available inherently, in a group or in the environment of which the individual is a part. Resources can be personal, such as good self-confidence and intellectual capacity. Resources available in the environment can be social support from someone one trusts, such as family, friends, health personnel and God.

*Meaningfulness* covers if something is worth engaging in and fighting for. Hence, the meaningfulness component includes motivational aspects for action, which presupposes comprehensibility and manageability. These three components overlap and interact (Antonovsky, 1987).

GRR and SRR are key resources for SOC in the salutogenic theory. Resistance represents what is difficult and challenging in an individual’s life (Antonovsky, 1987). In an NH context, resistance includes losses, ailments, symptom severity, loneliness, existential distress, etc. Resistance resources exist at the individual, group (family, peers), sub-culture and whole society levels. Accordingly, the nature of these resources is different: genetic and constitutional, psychosocial, cultural and spiritual, material and preventive health orientation (Haugan and Eriksson, 2021b). GRRs are resources at the disposal of both external and internal characters. An NH resident’s internal resources include the characteristics/abilities/properties of the person that make it easier to adapt to institutionalized NH life and cope with the finality of one’s life; e.g. a set of meaningful and coherent life experiences that in turn create a strong SOC, a strong faith in God, etc. (Espnes *et al.*, 2021; Haugan, 2021b; Haugan and Eriksson, 2021d). External GRRs involve social support by the NH resident’s family, friends and peers (Espnes *et al.*, 2021; Haugan and Eriksson, 2021d). In the NH context, specific resources (SRRs) could be the nurse–patient interaction, the provision of a supportive and physical environment along with specific interventions aimed at enhancing wellbeing and the joy of life (Andre et al., 2020, 2021, 2022). GRRs represent resources of broad benefit for efficient management of stressors (Espnes *et al.*, 2021; Haugan and Eriksson, 2021d); SRRs exemplify situation-specific benefits when approaching special stress situations, such as the experience of pain, fatigue, dyspnoea, existential loneliness and depressive symptoms (Espnes *et al.*, 2021). The salutogenic theory emphasizes ego identity and social support as critical GRRs (Lindström and Eriksson, 2010). In the NH context, the nurse–patient interaction, along with social support from family/friends, signify key salutogenic resources (Haugan and Eriksson, 2021c), representing crucial GRRs/SRRs in the long-term NH population.

Antonovsky developed the Orientation to Life Questionnaire (OLQ), which is widely used to assess SOC in various populations (Moksnes, 2021). The OLQ is available in two versions: one consisting of 29 items (OLQ-29) and the other is an abbreviated version consisting of 13 of the 29 items (OLQ-13). Each item is an indicator of one of the three dimensions of comprehension, manageability and meaningfulness. The responses are graded on a scale between 1 and 7. A low SOC indicates a low coping capacity, and a high score reflects better coping in the face of challenges (Antonovsky, 1987). Evidence demonstrates that strengthening an individuals’ SOC equates to promoting their health (Lindström and Eriksson, 2010; Hojdahl *et al.*, 2015; Birkeland *et al.*, 2017; Drageset *et al.*, 2020). Therefore, a vital question is how staff nurses can facilitate comprehensibility, manageability and meaningfulness among NH residents. Based on the OLQ-13, this article discusses the meaning of the OLQ items for NH residents’ wellbeing, exemplifying with OLQ-13 items how health professionals can promote NH resident’s SOC and thereby wellbeing.

Since SOC is highly correlated with wellbeing (Moksnes, 2021), older individuals’ perceived comprehensibility, manageability and meaningfulness are indicators of wellbeing and a good life (Antonovsky, 1987; Strandmark, 2006; Hojdahl *et al.*, 2015; Birkeland *et al.*, 2017; Bringsvor *et al.*, 2019; Drageset *et al.*, 2020). Proper NH care includes attention and a variety of approaches to promote mental, emotional, spiritual/existential and physical health and wellbeing. Thus, a shift from a pathogenic perspective focusing solely on diseases and losses to a salutogenic resource-oriented focus on supporting wellbeing and a meaningful everyday life seems necessary (Haugan, 2014b, 2021a; Patomella *et al.*, 2016). Consequently, NH residents’ perceived SOC, GRRs/SRRs and nurse–patient interaction are vital aspects of salutogenic NH care that promotes wellbeing and health.

This article elaborates and discusses how the salutogenic health theory involving NH residents’ SOC, GRRs and SRRs can be utilized to promote wellbeing among NH residents. Regarding residents’ SOC, items from Antonovsky’s OLQ-13 provide examples of vital approaches in salutogenic NH care.

## DISCUSSION

Having several chronic diseases causing different disabilities and the need to move into an NH for 24-h care is often a threatening life experience for people and is a demanding job for health care professionals. Therefore, using the SOC concept to make the NH context and its culture consistent as far as possible, with underload–overload balance, and participatory for residents, health care staff and visitors, could be an adequate argument and way to make NHs generally more salutogenically driven. Developing more salutogenic ‘standards’ and making the NH institutional contexts more salutogenic are possible. Even though Antonovsky assumed that one’s SOC cannot be radically transformed, he left it open that the SOC could be shaped and strengthened, so that it can push people towards health and wellbeing (Seah *et al.*, 2021). Therefore, with reference to NH residents’ situation, identifying GRRs and SRRs to improve their perceived meaningfulness, comprehensibility and manageability could become an explicit goal of salutogenic NH care. In the NH context, the health professionals are SRRs relieving residents’ ailments such as pain, constipation, sleeplessness and depressive symptoms, as well as providing specific tools supporting the resident’s coping and adapting abilities and thus autonomy; such tools may also be seen as SRRs.

### Strengthening comprehensibility

It is often challenging to cope with life changes due to illness and disability. The individual’s mastery depends on how they understand what is happening to them here and now. Table 1 lists the five OLQ items covering comprehensibility; these involve experiences of being surprised by the behaviour of people whom you think you know well, finding oneself in an unfamiliar situation, having mixed-up feelings and feelings that one would rather not have. The last of these five items (OLQ-11) has shown low validity among NH residents (Drageset and Haugan, 2016); therefore this item is not elaborated on here.

**Table 1:** OLQ items covering comprehensibility

Item no.	Description OLQ-13
2	‘Has it happened in the past that you were surprised by the behaviour of people whom you thought you knew well?’
6	‘Do you find that you are in an unfamiliar situation and do not know what to do?’
8	‘Do you have very mixed-up feelings and ideas?’
9	‘Does it happen that you have feelings inside you would rather not feel’
11	‘When something happened, have you generally found that…you overestimated or underestimated its importance… vs…. you saw things in the right proportion?’

NH residents need help with dressing, eating, toileting, going to bed, symptom management, etc.; thus, they might feel that they must completely hand themselves over to the nurses. Moreover, as a result of numerous losses, these individuals have had to leave their home and move into the institutionalized context of an NH. Daily life in an NH involves being exposed to specific routines and practices by others, as well as living close to strangers. Accordingly, NH residents may find themselves in an unfamiliar situation (OLQ-6), feeling helpless, unpowered, useless or even objectified. The nurses are key persons in this life situation; nurses who unsolicited utilize the nurse–patient interaction are in a position to offer meaningful contact, involving listening and acknowledging the resident’s experiences of his/her situation. Meaningful interaction accompanied by instrumental and emotional support are significant salutogenic resources (GRRs) for wellbeing (Espnes *et al.*, 2021; Haugan and Eriksson, 2021a,d). Accordingly, facilitating regular appointments with family, relatives and friends, either physically or by videoconference, along with offering books and opportunities to watch TV together with others, are vital to enhance NH residents’ wellbeing (Tsai and Tsai, 2011). Furthermore, the nurse–patient interaction should be refined to identify the resident’s GRRs and SRRs. What is important to the individual? What is on the resident’s mind? Which internal and external resources are at the individual’s disposal? Mapping the resident’s GRRs and SRRs is a crucial aspect of salutogenic NH care as well as the central question in PC.

Moreover, as an essential SRR in the NH context, the nurse–patient interaction is shown to be significant for NH residents’ experience of dignity, being understood, respected and valued (Haugan, 2021b). According to Kierkegaard (Kierkegaard, 1994): ‘If one is truly to succeed in leading a person to a specific place, one must first and foremost take care to find him where he is and start from there. This is the secret of the art of helping.’ (Kierkegaard, 1994). Nurses in NHs should explore how residents perceive their interaction with others; that is, letting the resident talk about being surprised by the behaviour of people whom they know well (OLQ item 2), having mixed-up feelings (OLQ item 8) or internal feelings (OLQ item 9), simply about what’s on their mind. ‘Person-oriented professionalism’ is the fundament of nurse–patient interaction, representing the core of nursing care; health professionals combine professional and human skills in their interaction with residents, which is based on the resident’s life-world [(Martinsen, 2006), pp. 73–78; Ellingsen, 2015]. Nurse–patient interaction includes both what is said and the unsaid, the tone of the speech conveys busyness or calmness, whether the attention is directed towards the resident or not, confirming the residents’ value and self-worth or not (Ellingsen, 2015; Ellingsen *et al.*, 2015). NH residents expect and need the nurses to be caring. However, sometimes they feel surprised by the nurses’ behaviour, as well as disappointed, sad and miserable (Haugan, 2021a). Nursing care providers must be aware that their attitude, appearance and behaviour are interpreted as a confirmation of the resident’s worthiness or worthlessness (Hedelin and Jonsson, 2003; Lundgren and Berg, 2011; Haugan et al., 2013, 2016; Haugan, 2014d). How the nurse is present together with the NH residents, looking and listening have been demonstrated to have a significant impact on anxiety, depressive symptoms, hope, meaning in life, inter- and intrapersonal self-transcendence, joy of life, loneliness and SOC in the NH population (Haugan et al., 2012, 2013, 2016, 2021; Haugan, 2013, 2014d; Drageset *et al.*, 2020; Reed and Haugan, 2021). The difference between feeling seen or overlooked, understood or misunderstood, has decisive implications for whether the individual feels trust or distrust in the nurses, as well as a sense of value and dignity, or unworthiness (Drageset and Ellingsen, 2016). Consequently, NH residents’ feelings are significantly influenced by the qualities embedded in the nurse–patient interaction, positively or negatively. Nursing professionals should be aware of their position, their power and that their relational behaviour may be a health-promoting SRR or be offensive and detrimental to the resident’s health and wellbeing.

### Strengthening manageability

Manageability involves using available GRRs/SRRs to promote health. Table 2 lists the four OLQ items covering manageability; these involve feelings such as being disappointed by people whom you counted on, being treated unfairly, being a loser in certain situations, along with keeping one’s feelings under control. OLQ item 13 has shown low validity and reliability among NH residents (Drageset and Haugan, 2016), therefore we omitted this item in the discussion.

**Table 2:** QLQ items covering manageability

Item no.	Description OLQ-13
3	Has it happened that people whom you counted on disappointed you?
5	Do you have the feeling that you are being treated unfairly?
10	Many people, even those with a strong character, sometimes feel like losers in certain situations. How often have you felt this way in the past?
13	How often do you have feelings that you are not sure you can keep under control?

NH residents present a wide range of ailments such as pain, dyspnoea, sleeplessness, loss of appetite, along with existential issues such as meaninglessness, boredom (Slettebo *et al.*, 2017; Rinnan *et al.*, 2022), existential loneliness and depression (Emilsdóttir and Gústafsdóttir, 2011; Beerens *et al.*, 2013; Erdal *et al.*, 2017; Campbell *et al.*, 2018). Moreover, living in an institutionalized setting such as an NH influences the residents’ QoL and wellbeing (Barca *et al.*, 2009; Haugan, 2014a; Rinnan *et al.*, 2021). Consequently, feeling like a loser (QLQ-10) may follow losses of functionality and being dependent on help from others for activities of daily living, such as toileting and eating. Resulting from this dependency on others, NH residents are a particularly vulnerable population and are therefore prone to feelings of being treated unfairly (QLQ-5). However, emotions can provide access to the significance of a situation for the resident and should be respected. In this way, emotions can be a gateway to early identification of ailments and thereby to promote wellbeing. Hence, there is much to cope with: accepting and adapting have been shown to be key individual GRRs in this situation (Haugan et al., 2012, 2016, 2022; Haugan, 2013; Reed and Haugan, 2021). Accordingly, facilitating and supporting NH residents’ acceptance of and adaption to their current functionality and life situation will boost their sense of manageability; manageable challenges can lead to a feeling of mastery.

Saunders emphasized that the patient and the family should be a ‘unit of care’ (Saunders, 2006). This refers to the importance of involving relatives. The NH residents’ condition and suffering have an impact on the people close to them, which means that the residents’ suffering is also the families’ suffering. Such a relationship may represent a central GRR. However, generally, NH residents’ social network is limited; they have been retired for many years, have left their home and familiar surroundings, and often their friends and spouse have passed away (QLQ-3). Counteracting disappointment, social isolation and loneliness are key in enhancing wellbeing among NH residents. Most frequently, NH staff are their helpers and main supporters. Accordingly, the health professionals must initiate social arenas based on reciprocity, trust and respect (Haugan *et al.*, 2020b; Drageset and Haugan, 2021; Haugan, 2021b). Consequently, the manner in which the individual is cared for and supported has a significant impact on their wellbeing (Haugan, 2021a). Confidence in the nurses and their capacity to solve difficult situations in the NH daily life is an important aspect of manageability. In promoting resident’s manageability, it can therefore be crucial to facilitate good conversations/companionship with health professionals or those in the resident’s network whose presence is health promoting (Haugan, 2013; Haugan *et al.*, 2013).

### Strengthening meaningfulness

Living in a meaningless situation means that resistance resources are not being used to promote health and wellbeing. Table 3 lists the four OLQ items covering meaningfulness. These include feeling that you do not care about what is going on, along with having or not having clear goals or purpose. Everyday activities are a source of pleasure/satisfaction or boredom, and a sense of meaninglessness in daily life.

**Table 3:** QLQ items covering meaningfulness

Item no.	Description OLQ-13
1	Do you have the feeling that you really don’t care about what is going on around you?
4	Until now your life has had: no clear goals or purpose at all …vs…. very clear goals and purpose
7	Doing the things you do every day is… a source of deep pleasure and satisfaction vs… a source of pain and boredom
12	How often do you have the feeling that there is little meaning in the things you do in your daily life?

In NHs, the overall goal is that the residents experience meaningfulness, joy of life and wellbeing in their daily life (Haugan, 2014b; Rinnan *et al*., 2018, 2022). Meaningfulness constitutes the aspect of motivation involved in SOC: when experiencing meaningfulness the individual will come to influence his/her present situation (Haugan and Rannestad, 2016). Connectedness is the core of self-transcendence (Reed and Haugan, 2021) and significant for meaningfulness (Drageset *et al.*, 2017; Haugan and Dezutter, 2021). However, NH residents have left their home to stay in an institutional context together with strangers; existential loneliness and perceived meaninglessness are common (Haugan, 2014a, b,c; Larsson *et al.*, 2017; Dewitte *et al.*, 2019; Drageset and Haugan, 2021).

Consequently, salutogenic NH care should develop standards to assess loneliness and what could facilitate joy of life for the individual resident. Specific interventions aimed at wellbeing and enjoying life should be utilized, representing SRRs; these could include cultural events, providing contact with animals, children and nature. Or, for example, arranging for shopping at the NH, making Christmas cookies together, providing a café, a hairdresser and so on in the NH building, arranging a men’s club for men talk, a bridge group, etc. Along with such interventions, facilitating a sound ‘health promotive working culture’ as an essential SRR is vital; therefore, systematically working with the health care staff and the working culture is essential in salutogenic NH care.

Fighting for meaningfulness and wellbeing amid existential experiences may prove difficult. For some individuals, accepting death may provide a sense of inner peace and meaning [(Martinsen, 2012), p. 123]. Within the last stage of life, it is important to support NH residents’ acceptance of death as being imminent and just being present together with the residents [(Martinsen, 2012), p. 123]. Existential concerns may be prominent in the last phase of life. Thus, the core palliative question ‘what is important to you now?’ is an essential spiritual/existential issue (QLQ-1 and QLQ-4). Nevertheless, what is important varies among individuals. Hence, health professionals in NHs must develop meaningful dialogues with residents to explore this issue. Spirituality, a GRR as well as an SRR, is described as a way of being in the world in which a person feels a sense of connectedness to self, others and/or a higher entity, nature and having a sense of meaning in life beyond self, everyday living and suffering (Weathers *et al.*, 2016). Connectedness can be seen as a vital GRR as well as an SRR; hence, facilitating the different aspects of connectedness (self, others, nature, God) would enhance the resident’s SOC. Spiritual needs may vary over time; therefore, NH professionals must persistently heed residents’ spiritual needs, which should, according to the International Council of Nurses (Ross and McSherry, 2019), be included as part of salutogenic nursing care in general. However, sometimes, spiritual/existential needs become profound issues that must be addressed with tenderness to avoid violating the resident’s dignity (Drageset and Ellingsen, 2016). Needs for spiritual/existential care must be identified in a sensitive manner along with the resident’s and the relatives’ other needs. A strengthened SOC may result from supporting the resident’s value and uniqueness by protecting and strengthening the person’s dignity, identity and integrity (Weathers *et al.*, 2016; Sæteren and Nåden, 2021). It is important to focus on the present situation, the ‘here and now’: an example of relevant questions in this situation can be ‘What makes you experience meaning in life right now?’ Crucially, if nurses use the approaches discussed here, they must show that they are willing to spend time to listen, and that they are able to accommodate and accept the resident’s replies and emotional reactions.

Meaningful activities represent GRRs, providing NH residents with possibilities to alleviate social isolation, loneliness and adapt to chronic diseases and thereby provide a sense of meaningfulness. If experiencing little meaning in daily life (QLQ-12), feeling that one’s daily activities are a source of pain and boredom (QLQ-7), residents’ perceived dignity might suffer. Nurses should gather information about the resident’s preferences for participation in meaningful activities at the NH (Slettebo *et al.*, 2017). In PC, activities such as music therapy have a key role in facilitating wellbeing; such life-giving PC strategies (Perez-Eizaguirre and Vergara-Moragues, 2020) might be transferred to NH care. When utilizing music therapy, the individuals’ musical preferences should be emphasized (Tziraki *et al.*, 2020). Moreover, it is possible to motivate NH residents by offering meaningful activities such as joining in a book club and videoconferences (Tsai and Tsai, 2011; Mondaca *et al.*, 2019), creating an audiobook club; and making practical arrangements for the residents to listen to audio books. Such activities may alleviate uncertainty, strengthen continuity, give a sense of control, maintain their knowledge and alleviate symptoms of depression and loneliness (Tsai and Tsai, 2011). However, to succeed, health professionals must identify and encourage the residents’ resources and establish companionships. Such enablement needs to be actively upheld by the NH community to build practices aligned with participation (Mondaca *et al.*, 2019).

## CONCLUSIONS

Generally, NH residents are characterized by frailty, impairment and symptom severity. Alleviation of existential and spiritual suffering along with pain and symptom management are central components of NH care; however, promoting health and wellbeing are equally important. Similar to assessing a resident’s symptoms and health condition, NH professionals should systematically identify the resident’s salutogenic resources, including GRRs, SRRs and SOC; these include the characteristics, abilities and properties of the resident as a person, the resident’s crucial life experiences, faith in God, persons of significance to the resident, etc. Assessing those aspects of the person requires a meaningful relationship based on dialogue. Consequently, salutogenic NH care is based on health-promoting nurse–patient interaction embedded in close and meaningful relationships to the residents. Based on this more holistic picture of both suffering and resources, salutogenic NH care can be developed to emphasize health promotion along with symptom management. Knowledge about SOC can help NH professionals to be aware of how residents comprehend, handle and find meaningfulness in this life situation, and thus support and facilitate SOC. Applying Antonovsky’s specific questions as a guide can help in identifying salutogenic resources and utilizing these to promote NH residents’ health and wellbeing. This requires that NH health professionals emphasize a holistic awareness of the resident’s bodily and mental experiences and use the nurse–patient interaction as a vital salutogenic resource in NH care.

### Clinical implications

This article discusses Antonovsky’s salutogenic health theory as a guide to promote wellbeing in NH residents. The help and support offered must be adapted to the NH resident’s individual needs, and opportunities to engage in meaningful activities should be provided. Nurses can promote residents’ SOC by facilitating meaningful conversations. This can be done by asking and identifying what is important and gives meaning to each NH resident; the answers should guide which activities to facilitate; this is, what makes sense in the specific life situation, what can the individual master, understand and handle. Future salutogenic interventions can involve various group sessions as well as individual encounters to facilitate social interaction and relieve loneliness. The palliative approach should be emphasized in NHs to include pain and symptom management along with existential care. However, most importantly, the salutogenic health theory should be implemented in NH care both as a philosophy and a practical guide to enhance residents’ SOC, health and wellbeing.
